# In Vivo Imaging Reveals a Pioneer Wave of Monocyte Recruitment into Mouse Skin Wounds

**DOI:** 10.1371/journal.pone.0108212

**Published:** 2014-10-01

**Authors:** Mathieu P. Rodero, Fabrice Licata, Lucie Poupel, Pauline Hamon, Kiarash Khosrotehrani, Christophe Combadiere, Alexandre Boissonnas

**Affiliations:** 1 Sorbonne Universités, UPMC Univ Paris 06, CR7, Centre d’Immunologie et des Maladies Infectieuses (CIMI), 91 Bd de l’hôpital, Paris, France; 2 INSERM, U1135, CIMI, 91 Bd de l’hôpital, Paris, France; 3 The University of Queensland, UQ Centre for Clinical Research, Brisbane, QLD, Australia; 4 The University of Queensland, UQ Diamantina Institute, Translational Research Institute, Woolloongabba, QLD, Australia; 5 CNRS, ERL, CIMI, 91 Bd de l’hôpital, Paris, France; Université de Technologie de Compiègne, France

## Abstract

The cells of the mononuclear phagocyte system are essential for the correct healing of adult skin wounds, but their specific functions remain ill-defined. The absence of granulation tissue immediately after skin injury makes it challenging to study the role of mononuclear phagocytes at the initiation of this inflammatory stage. To study their recruitment and migratory behavior within the wound bed, we developed a new model for real-time in vivo imaging of the wound, using transgenic mice that express green and cyan fluorescent proteins and specifically target monocytes. Within hours after the scalp injury, monocytes invaded the wound bed. The complete abrogation of this infiltration in monocyte-deficient CCR2^−/−^ mice argues for the involvement of classical monocytes in this process. Monocyte infiltration unexpectedly occurred as early as neutrophil recruitment did and resulted from active release from the bloodstream toward the matrix through microhemorrhages rather than transendothelial migration. Monocytes randomly scouted around the wound bed, progressively slowed down, and stopped. Our approach identified and characterized a rapid and earlier than expected wave of monocyte infiltration and provides a novel framework for investigating the role of these cells during early stages of wound healing.

## Introduction

Skin wound healing is a complex multistep process [1]. Seconds after injury, activated endothelial cells and platelets are helping to limit hemorrhage. The recruitment of inflammatory cells to the wound area follows, mostly neutrophils and, later on, cells belonging to the mononuclear phagocyte system (MPS), such as monocytes and macrophages. The subsequent recruitment of endothelial cells and fibroblasts promotes the formation of new tissue beneath the scab, called granulation tissue, which serves as a scaffold for the cells involved in wound healing. During its last stage, most of the cells that constitute the granulation tissue either undergo apoptosis or exit the cell. Finally, the remaining cells and extracellular matrix constitute the scar.

Macrophages are involved in all stages of skin wound healing [2]. Their depletion during the early stage of acute healing severely impairs its speed and quality [3], [4], [5], [6], while their depletion at later stages produces less serious effects, mostly on angiogenesis and fibrosis [5].

Recently, a polychromatic flow cytometry approach has made it possible to isolate and characterize the phenotype of wound-associated macrophages (WAMs) from excisional wounds. We and others have defined subsets of WAMs based on their phenotype and transcriptomic profile [7], [8], [9]. These studies have highlighted macrophage heterogeneity, which reflects the change in their functions during the course of healing; the macrophages switch from inflammatory populations to populations with proangiogenic/repair properties. This maturation is critical for proper wound healing [10] and can be polarized to restore defective healing in diabetic mice [9], [11].

WAMs arise primarily from circulating mature monocytes that have infiltrated the wound bed [9]. Two subsets of monocytes have been identified according to their expression of the lymphocyte antigen 6C (Ly6C) and two chemokine receptors, CCR2 and CX3CR1. CSF1R^+^Ly6C^high^CCR2^high^CX3CR1^low^ are inflammatory monocytes, while CSF1R^+^Ly6C^low^CCR2^neg^CX3CR1^high^ cells are the so-called resident monocytes. Expression of the macrophage colony stimulating factor receptor (CSF1R) is a hallmark of monocytes. It is acquired during ontogeny, and its expression is conserved on circulating monocytes and on those beginning to infiltrate tissue [12], [13]. CX3CR1 and CCR2 are the main chemokine receptors expressed by monocytes [14]; these receptors control their egress from the bone marrow and infiltration into inflamed tissue [15]. The intensity of their expression allows discrimination between monocyte subsets, although other phenotypic markers, such as Ly6C or 7/4, have commonly been used to define inflammatory and resident monocytes.

Despite significant progress in characterizing the involvement of mononuclear phagocytes (MPs) in the various acute and delayed stages of wound healing in recent years, the recruitment and the function of monocytes during the first hours after injury remain to be clearly defined in mammals. Current understanding of the process suggests that neutrophils colonize the wound first, probably as early as 2 hours after the injury [16], and are followed by monocyte recruitment, which starts 24 to 48 hours after injury [17]. In some models of inflammation, however, CX3CR1^+^ Ly6C^low^ monocytes are known to infiltrate inflamed tissue within hours and initiate subsequent immune responses [18]. In addition, CCR2^+^ monocytes may promote neutrophil extravasation [19], [20]. Reconciling these data requires further attention to the kinetics of monocyte infiltration into inflamed tissue during the earliest steps of the process.

To evaluate monocyte recruitment mechanisms in skin wound healing, we combined two transgenic systems: C*x3cr1* ^gfp/+^ [21] and *Csf1r*-Gal4VP16/UAS-ECFP (MacBlue) [22] and performed in vivo real time imaging.

In this study, we characterized the transient wave of inflammatory monocyte recruitment that occurred rapidly, concomitantly with neutrophil recruitment, during the first 4 hours after skin wound injury. We show that contrary to the extravasation mechanisms generally described, monocytes infiltrated the wound through active release from areas of microhemorrhage. Monocytes randomly migrated through the collagen network of the wound bed and subsequently stopped. These data open new perspectives in understanding the involvement of these cells in the outcome of wound healing.

## Materials and Methods

### Mice

*Cx3cr1*-GFP-Kin (*Cx3cr1* ^gfp/+^) and *Csf1r*-Gal4VP16/UAS-ECFP (MacBlue) mice were intercrossed to generate *Cx3cr1* ^gfp/+^×*Csf1r*-Gal4VP16/UAS-ECFP mice here named MacBlue×Cx3cr1^gfp/+^. This new strain was bred in the Nouvelle Animalerie Commune animal facility at Pitié-Salpétrière Hospital. All experiment protocols were approved by the french animal experimentation and ethics committee “Comité d’éthique pour expérimentation animale Charles Darwin n°5, Paris” and validated by “Service Protection et Santé Animales, Environnement” with the number A-75-2065. Sample sizes were chosen to assure reproducibility of the experiments and according to the 3R of animal ethic regulation.

### Flow cytometry

Flow cytometry was performed with the FACScanto flow cytometer (BD, Franklin Lakes, NJ), DIVA Flow Cytometry acquisition software, and Kaluza Flow Cytometry analysis software (Beckman Coulter, Miami, FL). Blood was drawn and directly stained with antibodies by incubating 50 µL of blood with 1 µg/mL purified anti-CD16/32 (2.4G2, BD Biosciences) for 10 min at 4°C to block Fc-mediated binding and for an additional 20 min with the appropriate dilution of specific antibodies. The panel of antibodies used was: anti-CD11b (clone M1/70), anti-Ly6C (clone AL-21), and anti-Ly6G (clone 1A8), NK1.1 (clone PK136) (all Becton Dickinson, San Jose, CA). After incubation, cell suspensions were washed once in PBS 0.5% BSA EDTA 2 mM and were directly analyzed by flow cytometry. Absolute cell numbers were calculated by adding to each vial a fixed number (10,000) of nonfluorescent 10-µm polybead carboxylate microspheres (Polysciences, Niles, IL) according to the formula: number of cells = (number of acquired cells×10,000)/(number of acquired beads). The number of cells obtained for each sample was normalized per mL of blood.

### Multiphoton imaging

Intravital imaging was performed on the deep skin layers of mouse skulls. Briefly, mice were anesthetized intraperitoneally with a mixture of ketamine (90 mg/kg bw; Ketamin 10%, Bela-Pharm, Vechta, Germany) and xylazine (25 mg/kg bw; Rompun 2%, Bayer Health Care, Leverkusen, Germany). Hair in the neck and scalp were removed, and a full-thickness excisional wound was created down to the panniculus carnosus with a 4-mm sterile punch (Stiefel Laboratories, Research Triangle Park, NC). The frontoparietal skull area was immobilized and exposed with a custom-made stereotactic holder. Mouse temperature was monitored and maintained at 37°C.

For the two-photon laser scanning microscopy (TPLSM) set-up, we used a Zeiss LSM 710 NLO multiphoton microscope (Carl Zeiss, Germany) coupled to a Ti:Sapphire Crystal laser (Coherent Chameleon, Santa Clara, CA, which provides 140 fs pulses of NIR light, selectively tunable between 680 and 1080 nm), and an acousto-optic modulator to control laser power. The system included three external non-descanned detectors with a combination of two dichroic mirrors (565 nm and 690 nm) with 565/610 and 500/550 bandpass filters and a 485 lowpass filter which allowed the simultaneous recording of three fluorescent channels. The excitation wavelength was 810 nm. Cell motility was measured every 30 s by 5 consecutive 3-µm z spacing stacks (total 12-µm thickness) with a plan apochromat ×20 (NA = 1) water immersion objective.

ECFP^+^ cells were tracked over time with 3D automatic tracking including manual correction with Imaris software (Bitplane, Zurich, Switzerland). Only cells that could be tracked for more than 2 min were considered. The arrest coefficient was defined as the proportion of time each cell’s instantaneous velocity (calculated for every 30-s interval) was below 2 µm/min. Track straightness was defined as the ratio of the distance between the initial and the final positions of each cell to the total distance covered by the same cell. Straightness analysis excluded all cells with a mean velocity below 2 µm/min. 3D images were reconstructed by compiling the 5-µm z spacing stacks. The acquisition and analysis protocols for all experimental conditions to be compared were identical.

### Statistical analysis

All statistical analyses were performed with Graphpad Prism 6 (Graphpad, San Diego, CA). One way ANOVA followed by Bonferroni adjustement was used to compare monocyte and neutrophils infiltration throughout time. Mann-Whitney rank sum tests were used to compare the cytometric analyses of the numbers and frequencies of cell populations. Kruskal-Wallis tests, followed by Dunn's multiple comparison tests, were used to compare highly skewed distributions (typically, intravital analysis of cell behavior). Symbols used *, p<0.05; **, p<0.01 ***, p<0.001, ns = not significant).

## Results

### Monocyte wound infiltration was localized to the deep skin layer

**Figure 1:**
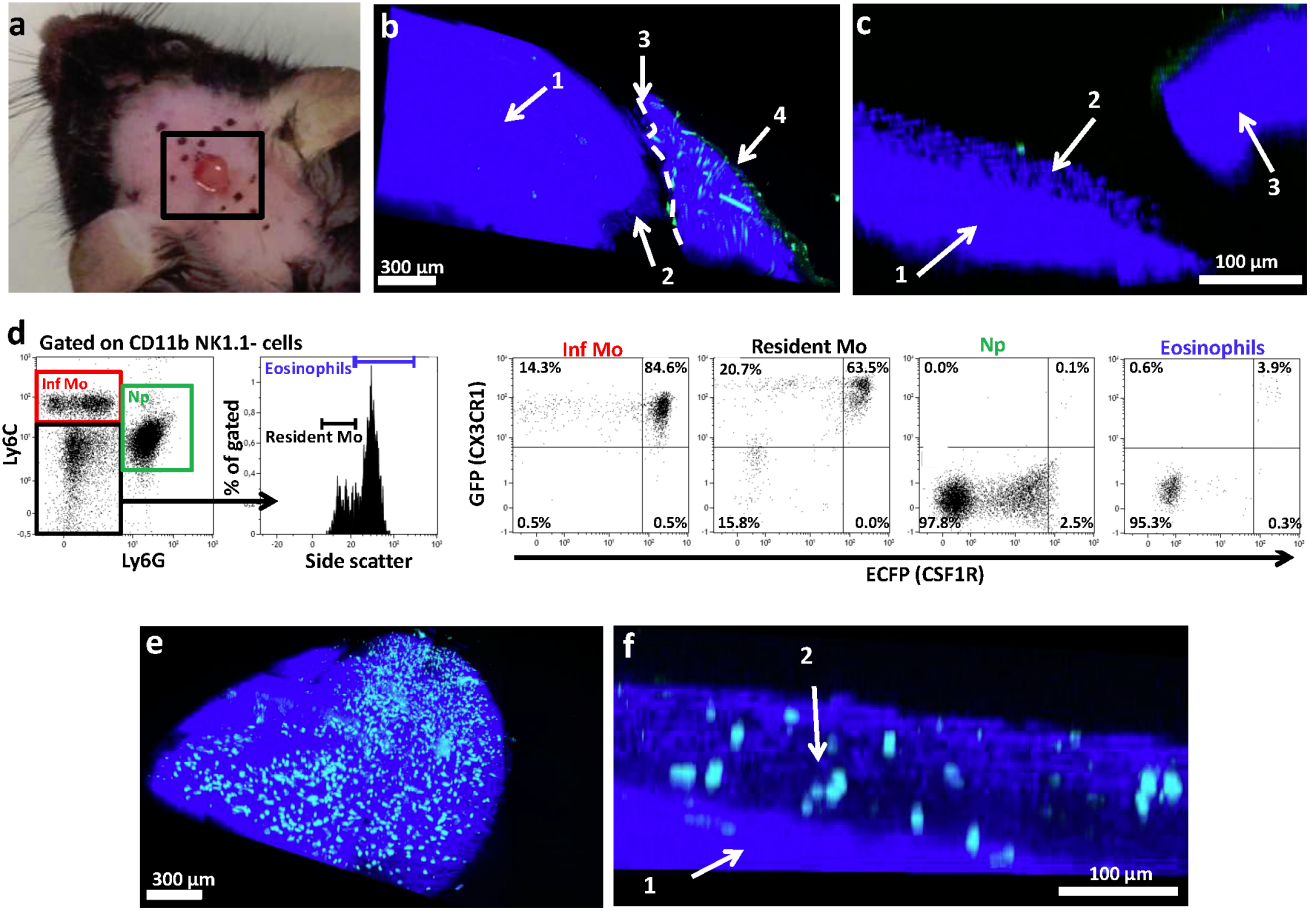
MP wound infiltration was localized to the deep skin layer. (**a**) Representative picture of skin wound excision. Pictures represent (**b**) front and (**c**) transversal two-photon laser scanning microscopy (TPLSM) 3D reconstruction of the wound site of a C57Bl6 mouse, 3 h after injury. The second harmonic generation (SHG) signal is in blue, and autofluorescent hairs in green. (**d**) Representative dot plot showing GFP and ECFP expression on distinct blood myeloid subsets from MacBlue×CX3CR1^gfp/+^ mice. (**e**) Front and (**f**) transversal TPLSM 3D reconstruction of the wound site in MacBlue×CX3CR1^gfp/+^ mouse, 4 h post-wounding. The SHG signal is in blue, the ECFP signal in cyan, and the GFP signal in green. Legends represent **1**: Skull; **2**: Deep skin layers; **3**: Wound edge; **4**: Skin. Inf Mo: Inflammatory monocytes; Np: neutrophils; Resident Mo: resident monocytes.

The absence of granulation tissue and the low quantity of cells available immediately after skin injury make it challenging to study this initial stage of leukocyte recruitment. Multiphoton in vivo imaging offers an interesting noninvasive alternative to flow cytometry and histology for monitoring leukocyte migration in its physiological environment at the cellular level. For this purpose, an excisional wound was made on each mouse scalp (**Fig. 1a**). Collagen detection by second harmonic signals distinguished bones from conjunctive tissues and skin, thereby mapping the structure of the wounded area (**Fig. 1b**): The front view shows a domed dense blue area corresponding to the mouse skull (1) and skin (4) with numerous green autofluorescent hairs. A transversal section of a 3D reconstructed image of the wound area reveals loose conjunctive tissue on the top of the skull, made up of the periosteum, hypodermis, and deep reticular dermis (2) (**Fig. 1c**). To track monocyte subsets by multiphoton imaging, we intercrossed *Cx3cr1* ^gfp/+^ mice with MacBlue mice to generate MacBlue×Cx3cr1^gfp/+^ mice expressing the specific fluorescent reporters ECFP and GFP in myeloid populations. This novel combination can distinguish 4 subsets among the well-characterized Ly6C^high^ and Ly6C^low^ monocytes [23] (defined as CD11b^+^ Ly6G^−^ and NK1.1^−^ cells) based on the expression levels of ECFP and GFP or the expression level of GFP alone (**Fig. 1d**). Indeed, 85% of blood Ly6C^high^ monocytes and 64% of the Ly6C^low^ monocytes coexpressed ECFP and GFP, while 14% and 21% respectively expressed only GFP. A small proportion of neutrophils (Np) expressed ECFP but no GFP (**Fig. 1d**). This system confers very bright combined ECFP and GFP fluorescence on monocytes compared to neutrophils (ECFP^low^) and NK subsets (GFP^+^). Four hours after the MacBlue×Cx3cr1^gfp/+^ mice were wounded, numerous GFP^+^ECFP^+^ cells had infiltrated the deep skin layer (2) (**Fig. 1e, f**). While detectable in the blood (**Fig. 1d**) and within uninjured skin (**Fig. S1**), no GFP^+^ECFP^−^ cells accumulated within the wound. Our system therefore provided the opportunity to study early myeloid cell infiltration within the wound at a cellular scale.

### The skin wound induced a rapid and transient wave of monocyte recruitment

**Figure 2:**
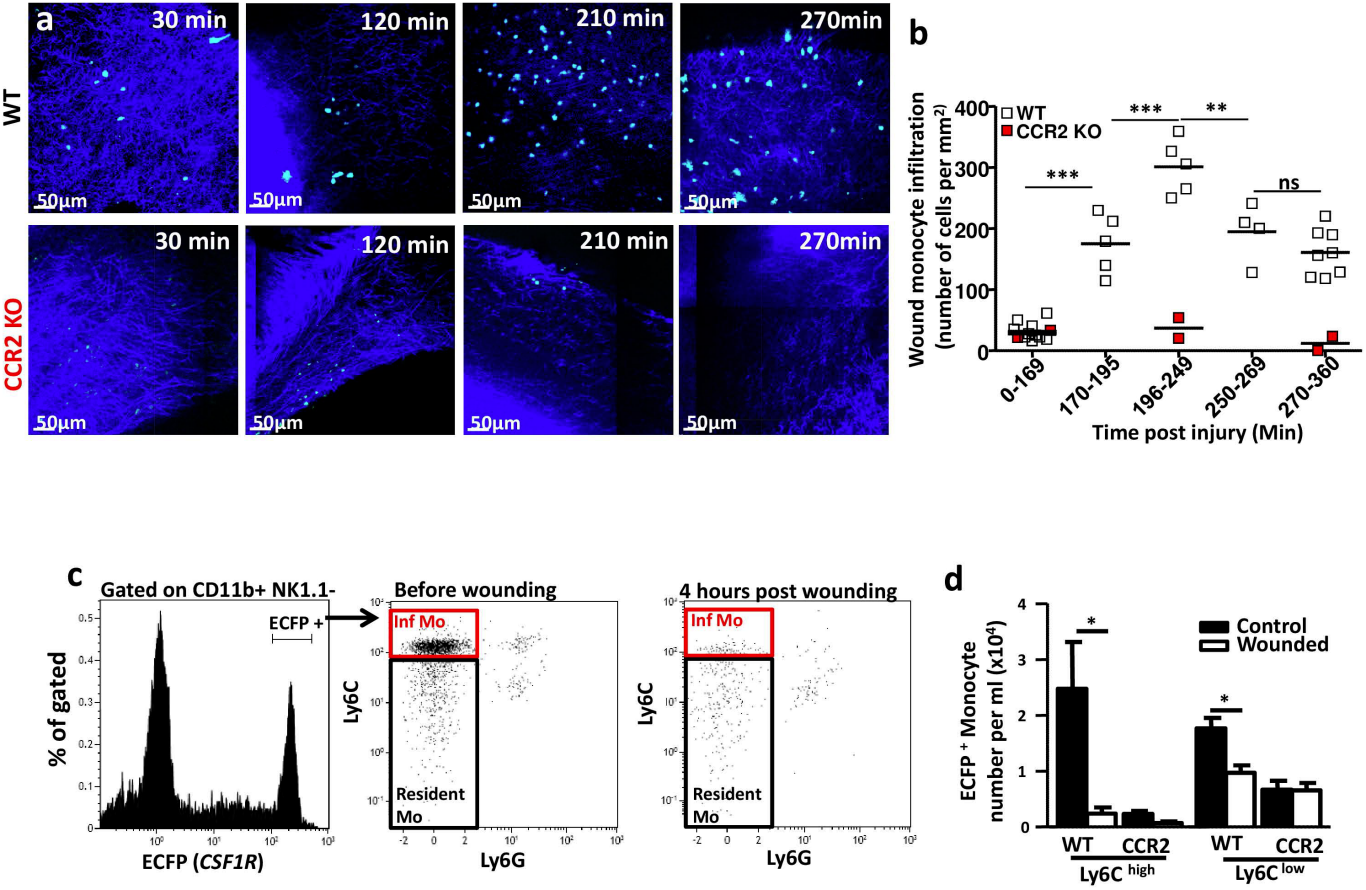
The skin wound induced a rapid and transient wave of monocyte recruitment. (**a**) Representative TPLSM picture of the wound deep skin layer from MacBlue×CX3CR1^gfp/+^ and MacBlue×CX3CR1^gfp/+^ CCR2^−/−^ mice at several time points after wounding. The SHG signal is in blue, the ECFP signal in cyan, and the GFP signal in green. (**b**) Quantification of ECFP^+^ cell density from TPLSM pictures taken 15 to 360 min post-wounding in MacBlue×CX3CR1^gfp/+^ and MacBlue×CX3CR1^gfp/+^ CCR2^−/−^ mice. Each dot represents a mean number of ECFP cells calculated from 30 to 50 different fields (100×100 µm) from 3–8 mice. Time points were compared using one way ANOVA followed by Bonferroni adjustment. (**c**) Representative flow cytometry dot plots of Ly6C and Ly6G expression by ECFP^+^ blood myeloid cells (gated on CD11b^+^, NK1.1^−^) from MacBlue×CX3CR1^gfp/+^ mice before and 4 hours after wounding. (**d**) Quantification of ECFP^+^ Ly6C^high^ inflammatory and Ly6C^low^ resident monocytes in the blood of MacBlue×CX3CR1^gfp/+^ and MacBlue×CX3CR1^gfp/+^ CCR2^−/−^ mice before and 4 hours after injury (n = 4–7 from 3 independent experiments). Inf Mo: Inflammatory monocytes; Resident Mo: Resident monocytes. *p<0.05; **: p<0.01; ***: p<0.001.

We aimed to evaluate the nature and the kinetics of this infiltrate in the first hours after the skin was wounded. ECFP^+^GFP^+^ cell density in the wound bed was evaluated by two-photon laser-scanning microscopy (TPLSM) images (**Fig. 2a** **upper panels**) at different times (up to 6 hours) after injury (**Fig. 2b**). A few ECFP^+^GFP^+^ cells were already present in the deep skin immediately after the injury, most likely tissue-resident cells. Their density remained constant for the first 170 min after injury (32±15 cells/mm^2^) and then rapidly accumulated by 250 min (up to 301±45 cells/mm^2^, p<0.001). From 250 to 270 min, the density of these cells dropped by 30% (194±48 cells/mm^2^, p<0. 01) and remained constant thereafter (161±38 cells/mm^2^, ns) (**Fig. 2b**).

To further investigate the phenotype of the ECFP^+^ monocytes infiltrating the wound, we performed intravital imaging of wounded MacBlue×Cx3cr1^gfp/+^CCR2^−/−^ mice (**Fig. 2a** **lower panels**). CCR2-deficient mice have a profound nearly total absence of peripheral Ly6c^high^ inflammatory monocytes, while the number of Ly6c^low^ monocytes is weakly affected [24]. TPLSM image quantification of CCR2-deficient mice displayed a complete absence of ECFP^+^GFP^+^ cell accumulation in the wound during the period studied (**Fig. 2b** **, red dots**). Accordingly, 240 min after the wounding of WT animals, the number of blood Ly6C^high^ cells decreased by 90%, while the number of ECFP^+^ Ly6C^low^ monocytes fell substantially less, by 46% (**Fig. 2c, d**). In CCR2^−/−^ mice, the wounding did not reduce the number of residual ECFP^+^ Ly6C^high^ and Ly6C^low^ blood monocytes (**Fig. 2d**). These observations strongly suggest that the transient wave of ECFP^+^ infiltrating cells was mostly composed of Ly6C^high^ monocytes.

### Monocytes infiltrated the wound bed concomitantly with neutrophils

**Figure 3:**
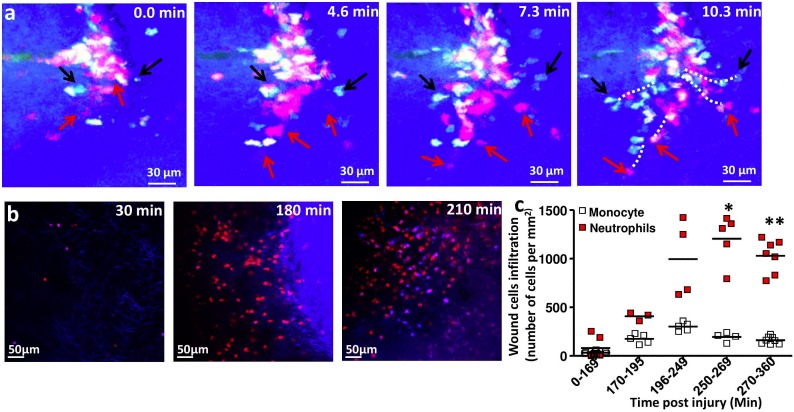
Monocytes infiltrated the wound bed concomitantly with neutrophils. (**a**): Time lapse picture series representing concomitant invasion of the wound bed by monocytes (black arrows) and neutrophils (red arrows). The SHG signal is in blue, the ECFP signal in cyan, and the PE signal in red. Representative track paths are represented by white dashed lines. (**b**) Representative TPLSM pictures of the wound deep skin layer from MacBlue×CX3CR1^gfp/+^ mice at several time points after wounding. Mice were injected intravenously with 10 µg of Ly6G-PE antibody before wounding. The SHG/ECFP signal is in blue, and the PE signal in red. (**c**) The graph shows the quantification of Ly6G^+^ and ECFP^+^ cell density from TPLSM pictures taken 15 to 360 min post-wounding in MacBlue×CX3CR1^gfp/+^ mice. Each dot represents a mean of number of ECFP^+^ cells calculated from 30 to 50 different fields (100×100 µm) from 3 to 8 different mice. Time points were compared using one way ANOVA followed by Bonferroni adjustment. *p<0.05; **: p<0.01.

The current model suggests that neutrophils are massively recruited during the hours that follow skin injury and that their infiltration precedes that of monocytes. We therefore compared the kinetics of monocyte and neutrophil infiltration by intravital imaging (made possibly by intravenous injection 10 min before wounding of a neutrophil-specific anti-Ly6G antibody conjugated with phycoerythrin). We observed neutrophils exiting vascular areas together with monocytes (**Fig. 3a** **and Video S1**). Neutrophils accumulated in the wound bed at the same time as initial monocyte recruitment took place (**Figure 3b, c**). But while monocyte density decreased progressively after 210 min, neutrophil density continued to increase until 270 min (170 min: 92±120 cells/mm^2^; 270 min: 1206±250 cells/mm^2^, p<0.05) and then stabilized (1029±170 cells/mm^2^, ns) (**Fig. 3c**). We thus concluded that monocyte and neutrophil infiltration of the wound bed took place concomitantly.

### ECFP^+^ monocytes infiltrated the wound bed through areas of microhemorrhages

**Figure 4:**
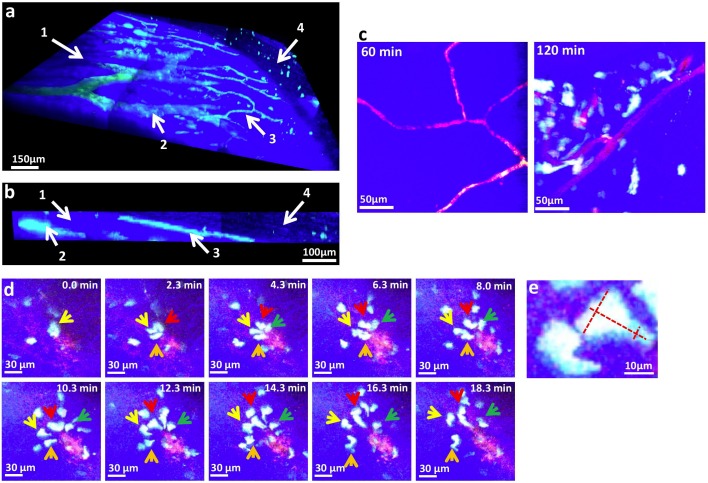
ECFP^+^ monocytes infiltrated the wound bed through areas of microhemorrhages. (**a**) Front and (**b**) transversal TPLSM 3D reconstruction of the wound site in MacBlue×CX3CR1^gfp/+^ mice 30 min post-wounding. 70 kDa fluorescein-dextran (green) was injected *i.v*. before the imaging session for vasculature staining. The SHG signal is in blue. Legends represent: **1**: Skull, **2**: Bone marrow vasculature, **3**: Superficial vasculature, **4**: Deep skin layer. (**c**) TPLSM pictures at different time point after wounding of MacBlue×CX3CR1^gfp/+^ mice. Vasculature staining was performed with high molecular weight (2 MDa) rhodamine-dextran (red). The ECFP signal is in cyan. (**d**) Time lapse image series representing monocyte (arrows) egress from a microhemorrhage area toward the wound bed. Colored arrows depict 4 distinct infiltrating monocytes. (**e**) Representative ECFP^+^ cell morphology during egress from the blood.

Numerous inflammatory molecules are released at wound sites [25] and promote the arrest of circulating cells in the endothelium and their extravasation toward the wound bed. We used our imaging approach to describe the process of monocyte entry into the deep skin layers of the wound. We first labeled blood vessels of the wounded region with (70-kDa)-fluorescein-dextran. Deep inside the skull (1), the large vessels were characterized by bone marrow sinusoids and collecting venules (2) (**Fig. 4a**). A transversal section of the 3D reconstructed image revealed a more superficial vascular network (3) at the interface of the skull and periosteum, but the deep skin layers were apparently devoid of vessels (4) (**Fig. 4b**).

To visualize the behavior of monocytes in the vicinity of the vasculature, we next used high molecular weight rhodamine dextran (2MDa) to detect vascular leakage. Up to 60 min post-wound, the subperiosteal vasculature was free of ECFP^+^ cells, but by 120 min, most vessels were covered with fluorescent cells (**Fig. 4c**). Numerous events of cell egress from the vasculature toward the wound site occurred at sites of diffuse rhodamine staining, which indicated massive vascular permeability or microhemorrhages (**Fig. 4d** **and Video S2**). During endothelial barrier crossing, leukocyte cytoskeletons are reorganized such that the leukocytes are spread out and split between two endothelial cells and thus create typical shape variations [26]. No such variation was observed. However, infiltrating cells displayed a very active motility pattern (**Fig. 4d**) rather than passive diffusion toward the matrix, and their morphology was characteristic of ameboid migration (**Fig. 4e**) [27]. These results suggest that the first wave of monocyte infiltration into the wound did not result from transendothelial migration but rather from direct crawling through vascular leakage.

### Wound-infiltrating monocytes migrated actively and then stopped within the matrix

**Figure 5:**
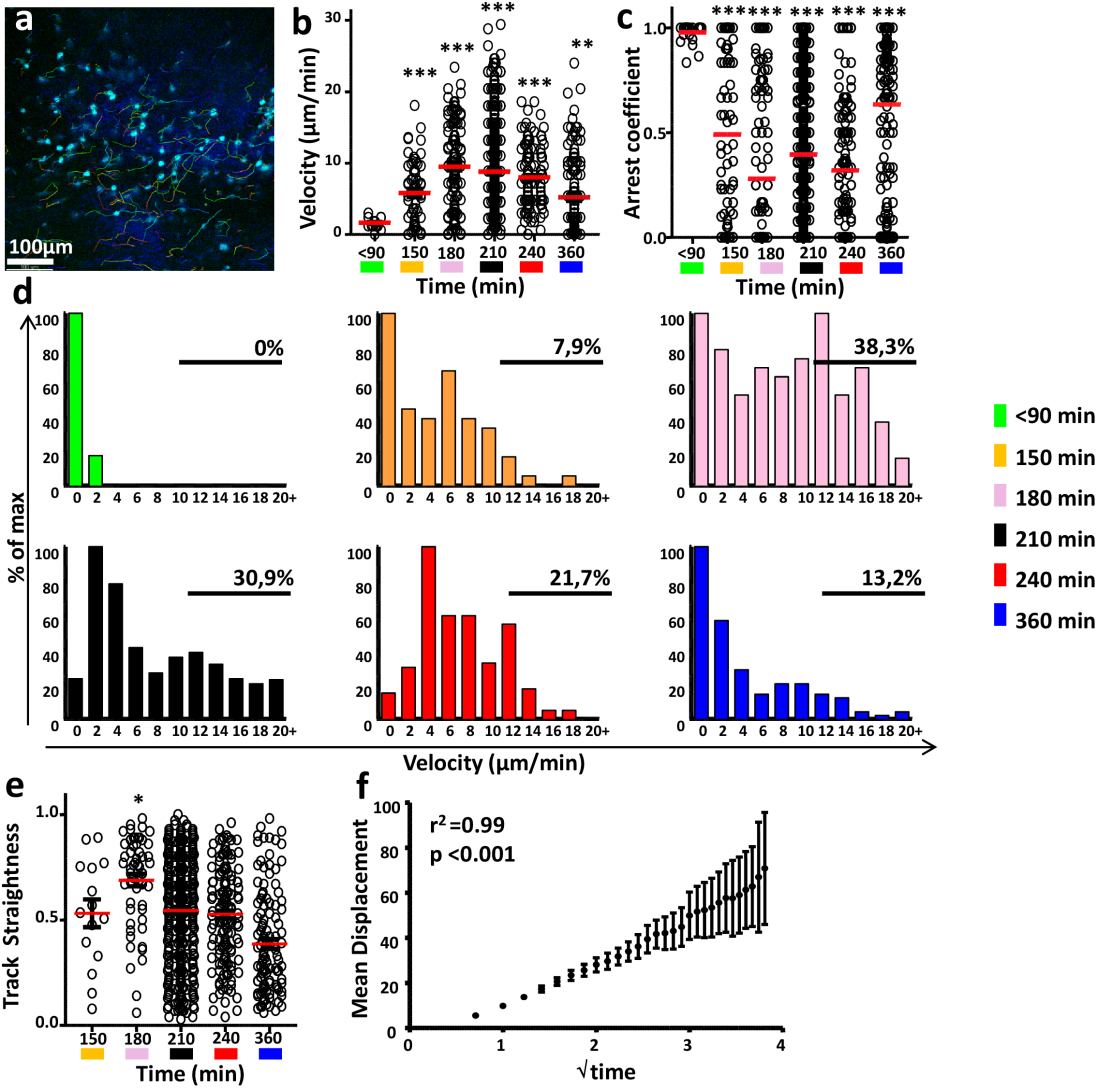
Wound-infiltrating monocytes migrated actively and then stopped within the matrix. (**a**) Representative TPLSM picture of monocyte migratory behavior 210 min after injury of MacBlue×CX3CR1^gfp/+^ mice. Track pathways of ECFP^+^ cells (cyan) are indicated. Quantification of ECFP^+^-cell mean velocity (**b**) and arrest coefficient (**c**) at indicated time range after injury. (**d**) Graphs represent cell frequency distribution as a function of their mean velocity at indicated time range after injury. The percentages of cells with a mean velocity higher than 12 µm/min are indicated. (**e**) Quantification of track straightness of ECFP^+^ cells at the indicated time range after injury. (**f**) Representation of the ECFP^+^-cell mean displacement as a function of the square root of time at 210 min after injury (all quantifications represent a pool of 27 to 781 cells from 3 to 6 different videos performed on 2 to 5 different mice). *p<0.05; ***p<0.001.

To address the mechanism of monocyte infiltration further, we performed time lapse imaging of the deep skin area at several time points after the mouse was wounded to study the monocyte migratory pattern. Cells were automatically tracked over time, and their mean velocity and arrest coefficient were evaluated up to 360 min after wounding (**Fig. 5a**). Cells present in the deep layers during the first 90 min after the wounding were sessile (**Video S3**), as expected for resident cells. ECFP^+^ cells had accumulated 90 min post-wounding, with apparent displacement within the wound (**Fig. 4b and c** **, Video S4**). The mean velocity of these cells increased from 90 to 180 min post-wounding (1.7±0.6 and 9.5±5.9 µm/min, p<0.001) and then progressively decreased over the remaining study period (to 5.2±4.3 µm/min, p<0.001). Consistent with the absence of significant displacement, the arrest coefficient of the resident cells imaged in the wound bed during the first 90 min after injury was high. A transient decrease in this coefficient was observed between 90 and 180 min (97±4% and 28±36%, p<0.001). Thereafter, the arrest coefficient of the newly infiltrating monocytes increased from 180 to 360 min (to 64±30; p<0.001).

To clarify the biological relevance of this behavior, we compared the frequency distribution as a function of the mean velocity at the different time points (**Fig. 5d**). A progressive increase in the proportion of monocytes with high-speed movement (>12 µm/min) between 90 and 210 min was observed; it correlated with the massive infiltration described above. At 210 min, the clearly bimodal cell distribution suggested differential cell behavior, with some moving at a high mean velocity and others with a high arrest coefficient. After 210 min, this bimodal distribution tended to vanish with a reduction in the proportion of fast-moving cells. This change was confirmed at 240 and 360 min, when cell movement was mainly arrested.

These results showed that the first cells infiltrating the wound had a low mean speed. After several minutes, the individual mean velocity increased with the number of infiltrating cells. Between 180 and 210 min, before the number of monocytes in the wound began decreasing (**Fig. 2**), their velocity slowed. Finally, by 240 min, when the monocyte numbers had fallen, the overall arrest coefficient of those remaining increased.

To determine whether the motion of cell infiltration tended to be directed, we evaluated the directionality of the individual monocyte trajectories by evaluating their track straightness (**Fig. 5e**). Mean track straightness increased between 150 and 180 min (from 0.5±0.2 to 0.7±0.2, p<0.05) and decreased progressively thereafter until the end of the experiment (to 0.4±0.2 p<0.001). This finding suggests that these cells displayed some directionality during the first minutes of infiltration and lost this directionality once inside the wound bed. The displacement calculated as a function of the square root of time at 210 min post-wounding (when monocytes stopped accumulating) indicated the very high linearity (r^2^ = 0.99; p<0,0001) (**Fig. 5f**) characteristic of random migratory patterns, as others have described [28], [29].

These findings led us to conclude that monocytes penetrated in a relatively directed manner toward the deep wound layers and progressed by random motion, invading the collagen matrix across the wounded area before arresting, most likely to exert specific functions.

## Discussion

Cells of the innate immune system are the first to be mobilized after skin is wounded. Small dysregulations of the inflammatory response often lead to chronic disorders and the failure of effective healing. A correct reading of the different steps of cellular infiltration during wound healing is thus essential to improve our understanding of wound-associated disorders and enable us to counteract them. Because the granulation tissue does not form in the first early minutes after the skin is wounded, isolating and characterizing the earliest infiltrate into the wound bed has presented quite a challenge. Thus, investigations of early immune cell recruitment mechanisms and kinetics in mammalian skin wounds in the first hours after their occurrence have not yet been adequately investigated.

For this study, we used the MacBlue×Cx3cr1^gfp/+^ mouse, a combined multiple fluorescent reporter transgenic model, to specifically label different monocyte populations and apply a new approach of real time in vivo multiphoton imaging to study cell dynamics during the early phase of recruitment into the wound bed before the generation of the granulation tissue. The advantage of this approach is that it provides resolution at the cellular level and thus makes it possible to track individual cells from different subsets according to the specificity of the fluorescent reporter used. Based on in vivo imaging at such a cellular resolution, Li et al showed that in newly injured zebrafish models, local resident macrophages and neutrophils are the first myeloid cells recruited, followed shortly after by circulating neutrophils, and later on by circulating monocytes [30].

Here, we have described an early and transient recruitment of monocytes during the first hours after mice are wounded. Current understanding holds that neutrophils are the first cells to infiltrate the inflamed tissue, followed by monocytes [31]. The specific staining of neutrophils and monocytes here allowed a clear identification of both populations and leads to the conclusion that the infiltration kinetics was similar for monocytes and neutrophils, although there were fewer monocytes. We observed that monocyte accumulation stopped after 250 min post-wounding, while neutrophils continued to accumulate until 270 min. Independent inflammatory models indicate that CCR2^+^ monocytes may promote neutrophil extravasation [19], [20] and thus confirm the importance of concomitant infiltration by the two populations. More recently, perivascular macrophages have been reported to be involved in this process during *Staphylococcus aureus* skin infection [32]. This primary process may be necessary for neutrophils to favor both the recruitment of subsequent waves of monocytes into inflamed tissues through promotion of monocytes arrested in activated endothelium and diapedesis [33]. We previously showed that once granulation tissue is formed a second wave of WAMs accumulated, starting by 24 h post-wounding and continuing for 5 days [9]. Together, these findings describe an organized sequence of cell-subset recruitment. Whether the initial wave of monocytes we described here is involved in the concomitant extravasation of neutrophils and the subsequent WAM wave requires further investigation.

The cell egress from the vasculature toward the wound bed in regions with rhodamine diffusion suggests that egress occurred in areas where vessel integrity was lost, due to either increased permeability or microhemorrhages. We observed here that monocytes used leaky areas of the vasculature to infiltrate the collagen matrix of the conjunctive tissue. Injury-induced vessel permeability is a transient event that may play a role in the first wave of monocyte recruitment, whereas the number of neutrophils keeps increasing after resolution of vessel permeability, as Kim et al have shown [16]. These observations suggest that neutrophils have complementary mechanisms of extravasation, independent of the vessel permeability that indirectly regulates the sequence of cell infiltration.

The density of cell infiltration and retention in the vicinity of blood vessels was consistent with the reduced number of blood monocytes and suggests that the first wave derived from circulating monocytes. The absence of monocyte recruitment in CCR2-deficient mice likely resulted from the reduced frequency of circulating inflammatory monocytes in this model, but the role of CCR2 in monocyte entry into the wound bed cannot yet be excluded [8].

The contribution of chemokine gradients to the migration pattern of leukocytes is difficult to address in vivo as it is now clear that migratory behavior is governed at least in part by haptotactic chemokine-driven attraction [34] and constrained motility, due to tissue architecture and barriers. In addition, in vivo work in zebrafish has suggested that soluble chemokine-dependent directed migration results in the deceleration of infiltrated cells as they near the chemokine source [35]. Our analysis of monocyte dynamics within the collagen matrix showed that the chemokine index (track straightness) increased between 90 and 180 min and thus suggested that motion was relatively directed at an early time point. The track straightness tended to diminish thereafter, as velocity fell and the arrest coefficient increased. The mathematical distribution of cell displacement as a function of the square root of time [29] showed that the motion of cells became random before it stopped. Our results point out an important change in the dynamics of monocytes once they reach the wound bed. Classically, leukocytes stop moving once activated, to exert their effector functions such as cytokine secretion, phagocytosis, or antigen recognition. Consistent with these previous observations, we can hypothesize that monocytes display high motility for a short time and then stop rapidly after activation to exert specific functions. The role of the pioneer wave of monocyte in wound healing remains to be determined. Previous report describes defective recruitment of inflammatory monocyte to skin wound at later time points in CCR2 -deficient mice. This defect was associated with altered wound healing; mostly on the angiogenesis aspect [8].

Overall, this study revealed and characterized an early, rapid wave of inflammatory monocyte recruitment within the first hours after an excisional skin wound. Given the importance of inflammation in controlling wound infection and closure, this novel aspect of wound-related inflammation provides a framework for investigating leukocyte migration, function, and cell-cell interaction in many settings, such as delayed wound healing or after therapeutic treatment.

## Supporting Information

Figure S1 **GFP+ ECFP- cells were detectable within the skin surrounding the wound.** Representative TPLSM pictures of superficial skin layer from MacBlue×CX3CR1^gfp/+^ mice at proximity of the wound edge. SHG signal is in blue, GFP signal is in green.
(PPTX)

Video S1 **Concomitant egress of neutrophils and monocytes from the vasculature.** TPLSM movie of the area by which monocytes and neutrophils exit the vasculature of a MacBlue×CX3CR1^gfp/+^ mouse 120 min after wounding. CFP^+^ monocytes are in cyan, neutrophils in red, and the bone and collagen matrix, which are visualized by second harmonic generation, are in blue.
(AVI)

Video S2 **Monocyte cell egress through microhemorrhages.** TPLSM movie of the area by which monocytes exit the vasculature of a MacBlue×CX3CR1^gfp/+^ mouse. CFP^+^ monocytes are in cyan, and the bone and collagen matrix, which are visualized by second harmonic generation, are in blue. 2 MDa rhodamine-dextran (red) is injected before imaging session to identify vasculature leakage (pale red).
(AVI)

Video S3 **Representative sessile behavior of CFP^+^ monocytes in the deep skin layer during the first 90 min post-wounding.** TPLSM movie of ECFP^+^ monocytes 30 min post-injury of a MacBlue×CX3CR1^gfp/+^ mouse. ECFP^+^ monocytes are in cyan; the bone and collagen matrix, which are visualized by second harmonic generation, are in blue.
(AVI)

Video S4 **Representative CFP^+^ monocyte infiltration into the deep skin layer.** TPLSM movie of ECFP^+^ monocytes 210 min post-injury of a MacBlue×CX3CR1^gfp/+^ mouse. ECFP^+^ monocytes are in cyan, and the bone and collagen matrix, which are visualized by second harmonic generation, are in blue. Cell tracks are visualized by colored lines.
(AVI)

## References

[1] Gurtner GC, Werner S, Barrandon Y, Longaker MT (2008) Wound repair and regeneration. Nature 453: 314–321. 18480812 10.1038/nature07039

[2] Rodero MP, Khosrotehrani K (2010) Skin wound healing modulation by macrophages. International Journal of Clinical and Experimental Pathology 3: 643–653. 20830235 PMC2933384

[3] Mirza R, DiPietro LA, Koh TJ (2009) Selective and specific macrophage ablation is detrimental to wound healing in mice. Am J Pathol 175: 2454–2462. 19850888 10.2353/ajpath.2009.090248 PMC2789630

[4] Goren I, Allmann N, Yogev N, Schurmann C, Linke A, et al (2009) A transgenic mouse model of inducible macrophage depletion: effects of diphtheria toxin-driven lysozyme M-specific cell lineage ablation on wound inflammatory, angiogenic, and contractive processes. Am J Pathol 175: 132–147. 19528348 10.2353/ajpath.2009.081002 PMC2708801

[5] Lucas T, Waisman A, Ranjan R, Roes J, Krieg T, et al (2010) Differential roles of macrophages in diverse phases of skin repair. J Immunol 184: 3964–3977. 20176743 10.4049/jimmunol.0903356

[6] Rodero MP, Legrand JMD, Bou-Gharios G, Khosrotehrani K (2013) Wound-associated macrophages control collagen 12 transcription during the early stages of skin wound healing. Experimental Dermatology 22: 143–145. 23278967 10.1111/exd.12068

[7] Mirza R, Koh TJ (2011) Dysregulation of monocyte/macrophage phenotype in wounds of diabetic mice. Cytokine 56: 256–264. 21803601 10.1016/j.cyto.2011.06.016

[8] Willenborg S, Lucas T, van Loo G, Knipper JA, Krieg T, et al (2012) CCR2 recruits an inflammatory macrophage subpopulation critical for angiogenesis in tissue repair. Blood 120: 613–625. 22577176 10.1182/blood-2012-01-403386

[9] Rodero MP, Hodgson SS, Hollier B, Combadiere C, Khosrotehrani K (2013) Reduced Il17a expression distinguishes a Ly6c(lo)MHCII(hi) macrophage population promoting wound healing. Journal of Investigative Dermatology 133: 783–792. 23235530 10.1038/jid.2012.368

[10] Mirza RE, Fang MM, Weinheimer-Haus EM, Ennis WJ, Koh TJ (2013) Sustained Inflammasome Activity in Macrophages Impairs Wound Healing in Type 2 Diabetic Humans and Mice. Diabetes. 10.2337/db13-0927 PMC3931398 24194505

[11] Mirza RE, Fang MM, Ennis WJ, Koh TJ (2013) Blocking interleukin-1beta induces a healing-associated wound macrophage phenotype and improves healing in type 2 diabetes. Diabetes 62: 2579–2587. 23493576 10.2337/db12-1450 PMC3712034

[12] Hume DA, MacDonald KP (2012) Therapeutic applications of macrophage colony-stimulating factor-1 (CSF-1) and antagonists of CSF-1 receptor (CSF-1R) signaling. Blood 119: 1810–1820. 22186992 10.1182/blood-2011-09-379214

[13] MacDonald KP, Rowe V, Bofinger HM, Thomas R, Sasmono T, et al (2005) The colony-stimulating factor 1 receptor is expressed on dendritic cells during differentiation and regulates their expansion. J Immunol 175: 1399–1405. 16034075 10.4049/jimmunol.175.3.1399

[14] Geissmann F, Jung S, Littman DR (2003) Blood monocytes consist of two principal subsets with distinct migratory properties. Immunity 19: 71–82. 12871640 10.1016/s1074-7613(03)00174-2

[15] Jacquelin S, Licata F, Dorgham K, Hermand P, Poupel L, et al (2013) CX3CR1 reduces Ly6Chigh-monocyte motility within and release from the bone marrow after chemotherapy in mice. Blood 122: 674–683. 23775714 10.1182/blood-2013-01-480749

[16] Kim MH, Liu W, Borjesson DL, Curry FR, Miller LS, et al (2008) Dynamics of neutrophil infiltration during cutaneous wound healing and infection using fluorescence imaging. Journal of Investigative Dermatology 128: 1812–1820. 18185533 10.1038/sj.jid.5701223 PMC2617712

[17] Stramer BM, Mori R, Martin P (2007) The inflammation-fibrosis link? A Jekyll and Hyde role for blood cells during wound repair. Journal of Investigative Dermatology 127: 1009–1017. 17435786 10.1038/sj.jid.5700811

[18] Auffray C, Fogg D, Garfa M, Elain G, Join-Lambert O, et al (2007) Monitoring of blood vessels and tissues by a population of monocytes with patrolling behavior. Science 317: 666–670. 17673663 10.1126/science.1142883

[19] Wang B, Zinselmeyer BH, Runnels HA, LaBranche TP, Morton PA, et al (2012) In vivo imaging implicates CCR2(+) monocytes as regulators of neutrophil recruitment during arthritis. Cell Immunol 278: 103–112. 23121982 10.1016/j.cellimm.2012.07.005 PMC3760198

[20] Kreisel D, Nava RG, Li W, Zinselmeyer BH, Wang B, et al (2010) In vivo two-photon imaging reveals monocyte-dependent neutrophil extravasation during pulmonary inflammation. Proc Natl Acad Sci U S A 107: 18073–18078. 20923880 10.1073/pnas.1008737107 PMC2964224

[21] Jung S, Aliberti J, Graemmel P, Sunshine MJ, Kreutzberg GW, et al (2000) Analysis of fractalkine receptor CX(3)CR1 function by targeted deletion and green fluorescent protein reporter gene insertion. Mol Cell Biol 20: 4106–4114. 10805752 10.1128/mcb.20.11.4106-4114.2000 PMC85780

[22] Ovchinnikov DA, van Zuylen WJ, DeBats CE, Alexander KA, Kellie S, et al (2008) Expression of Gal4-dependent transgenes in cells of the mononuclear phagocyte system labeled with enhanced cyan fluorescent protein using Csf1r-Gal4VP16/UAS-ECFP double-transgenic mice. J Leukoc Biol 83: 430–433. 17971498 10.1189/jlb.0807585

[23] Sunderkotter C, Nikolic T, Dillon MJ, Van Rooijen N, Stehling M, et al (2004) Subpopulations of mouse blood monocytes differ in maturation stage and inflammatory response. J Immunol 172: 4410–4417. 15034056 10.4049/jimmunol.172.7.4410

[24] Shi C, Pamer EG (2011) Monocyte recruitment during infection and inflammation. Nat Rev Immunol 11: 762–774. 21984070 10.1038/nri3070 PMC3947780

[25] Hubner G, Brauchle M, Smola H, Madlener M, Fassler R, et al (1996) Differential regulation of pro-inflammatory cytokines during wound healing in normal and glucocorticoid-treated mice. Cytokine 8: 548–556. 8891436 10.1006/cyto.1996.0074

[26] Imhof BA, Aurrand-Lions M (2004) Adhesion mechanisms regulating the migration of monocytes. Nat Rev Immunol 4: 432–444. 15173832 10.1038/nri1375

[27] Mogilner A, Keren K (2009) The shape of motile cells. Curr Biol 19: R762–771. 19906578 10.1016/j.cub.2009.06.053 PMC2864320

[28] Cahalan MD, Parker I (2008) Choreography of cell motility and interaction dynamics imaged by two-photon microscopy in lymphoid organs. Annu Rev Immunol 26: 585–626. 18173372 10.1146/annurev.immunol.24.021605.090620 PMC2732400

[29] Sumen C, Mempel TR, Mazo IB, von Andrian UH (2004) Intravital microscopy: visualizing immunity in context. Immunity 21: 315–329. 15357943 10.1016/j.immuni.2004.08.006

[30] Li L, Yan B, Shi YQ, Zhang WQ, Wen ZL (2012) Live imaging reveals differing roles of macrophages and neutrophils during zebrafish tail fin regeneration. J Biol Chem 287: 25353–25360. 22573321 10.1074/jbc.M112.349126 PMC3408142

[31] Mantovani A, Cassatella MA, Costantini C, Jaillon S (2011) Neutrophils in the activation and regulation of innate and adaptive immunity. Nat Rev Immunol 11: 519–531. 21785456 10.1038/nri3024

[32] Abtin A, Jain R, Mitchell AJ, Roediger B, Brzoska AJ, et al (2014) Perivascular macrophages mediate neutrophil recruitment during bacterial skin infection. Nat Immunol 15: 45–53. 24270515 10.1038/ni.2769 PMC4097073

[33] Soehnlein O, Zernecke A, Weber C (2009) Neutrophils launch monocyte extravasation by release of granule proteins. Thromb Haemost 102: 198–205. 19652869 10.1160/TH08-11-0720

[34] Weber M, Hauschild R, Schwarz J, Moussion C, de Vries I, et al (2013) Interstitial dendritic cell guidance by haptotactic chemokine gradients. Science 339: 328–332. 23329049 10.1126/science.1228456

[35] Sarris M, Masson JB, Maurin D, Van der Aa LM, Boudinot P, et al (2012) Inflammatory chemokines direct and restrict leukocyte migration within live tissues as glycan-bound gradients. Curr Biol 22: 2375–2382. 23219724 10.1016/j.cub.2012.11.018

