# Correction: In Vivo Imaging Reveals a Pioneer Wave of Monocyte Recruitment into Mouse Skin Wounds

**DOI:** 10.1371/journal.pone.0115508

**Published:** 2014-12-08

**Authors:** The PLOS ONE Staff

Figure S1 is incorrect. Please see the corrected Figure S1 here.

## Supporting Information

Figure S1 **GFP+ ECFP- cells were detectable within the skin surrounding the wound.** Representative TPLSM pictures of superficial skin layer from MacBlue ×CX3CR1^gfp/+^ mice at proximity of the wound edge. SHG signal is in blue, GFP signal is in green.
(PPTX)
