# Supplementary material for: In Vivo Imaging Reveals a Pioneer Wave of Monocyte Recruitment into Mouse Skin Wounds
Source: PLoS One. 2014 Oct 1;9(10):e108212. doi: 10.1371/journal.pone.0108212 (PMC4182700; doi:10.1371/journal.pone.0108212)
Supplement: Figure S1 — GFP+ ECFP- cells were detectable within the skin surrounding the wound. Representative TPLSM pictures of superficial skin layer from MacBlue×CX3CR1gfp/+ mice at proximity of the wound edge. SHG signal is in blue, GFP signal is in green. (PPTX) [file pone.0108212.s001.pptx]

## Slide 1
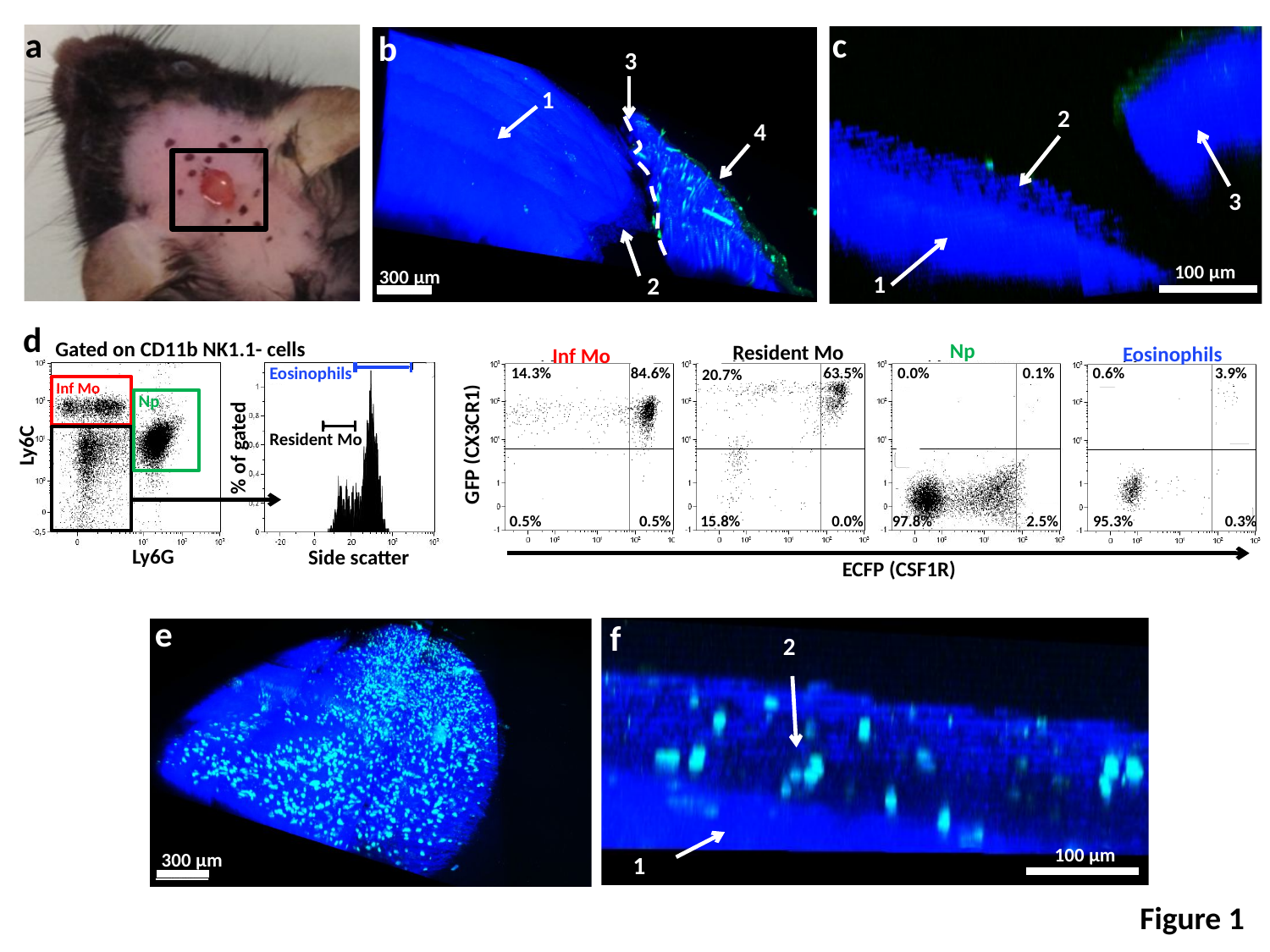

c
2
3
100 μm
1
a
b
3
1
4
300 μm
2
d
Gated on CD11b NK1.1- cells
Np
Resident Mo
Eosinophils
Inf Mo
Eosinophils
14.3%
84.6%
63.5%
0.0%
0.1%
0.6%
3.9%
20.7%
Inf Mo
Np
Resident Mo
GFP (CX3CR1)
Ly6C
% of gated
0.5%
0.5%
15.8%
0.0%
97.8%
2.5%
95.3%
0.3%
Ly6G
Side scatter
ECFP (CSF1R)
e
300 μm
f
2
100 μm
1
Figure 1

## Slide 2
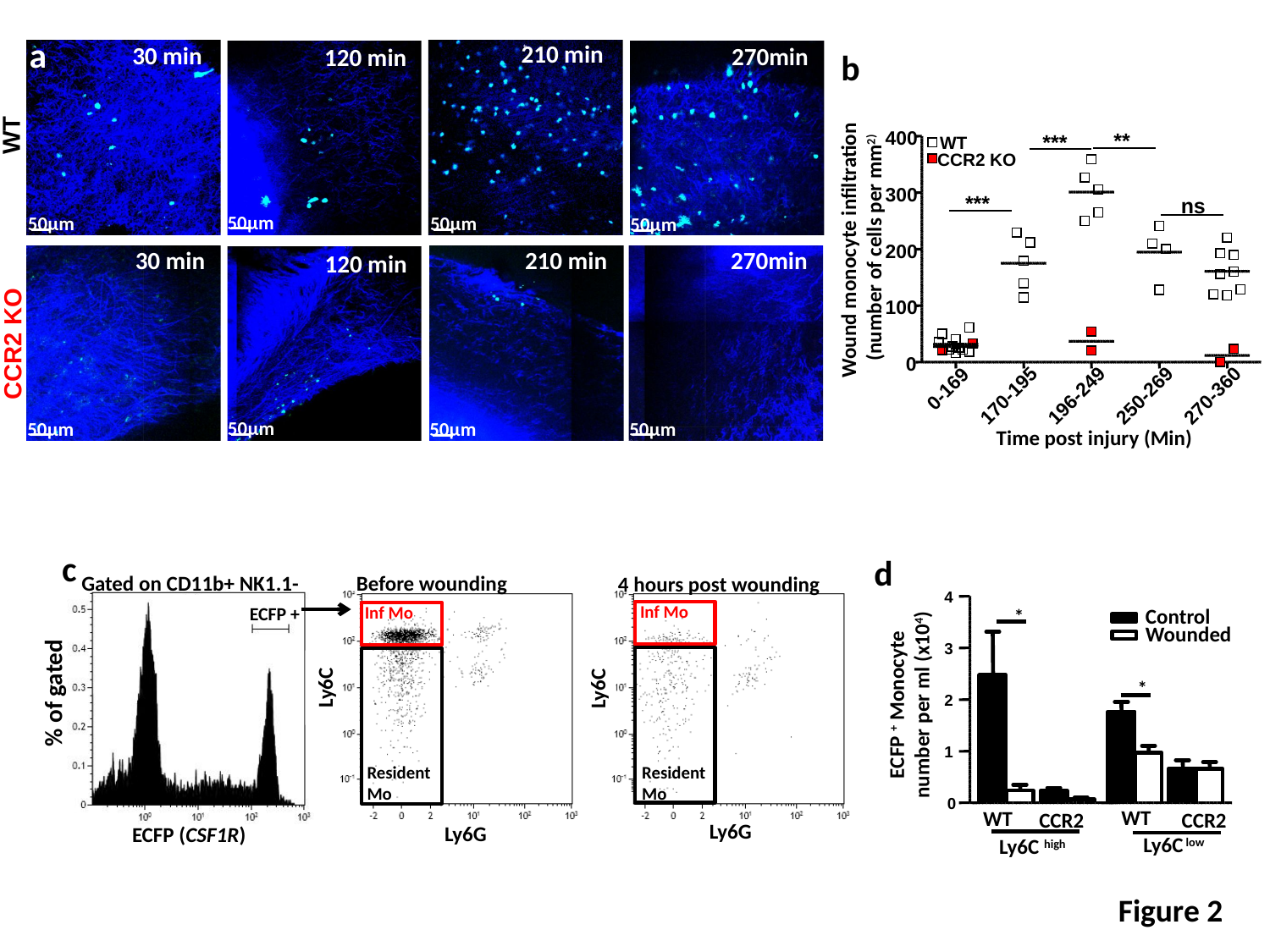

a
210 min
30 min
270min
120 min
WT
50μm
50μm
50μm
50μm
210 min
30 min
270min
120 min
CCR2 KO
50μm
50μm
50μm
50μm
b
**
***
400
WT
CCR2 KO
300
***
ns
Wound monocyte infiltration
 (number of cells per mm2)
200
100
0
0-169
170-195
196-249
250-269
270-360
Time post injury (Min)
c
d
Gated on CD11b+ NK1.1-
Before wounding
4 hours post wounding
 Ly6C
 Ly6G
 Ly6C
% of gated
Ly6G
ECFP (CSF1R)
4
Inf Mo
Inf Mo
ECFP +
Control
Wounded
*
3
*
Ly6C low
ECFP + Monocyte
number per ml (x104)
2
1
Resident
Mo
Resident
Mo
0
WT
WT
CCR2
CCR2
Ly6C high
Figure 2

## Slide 3
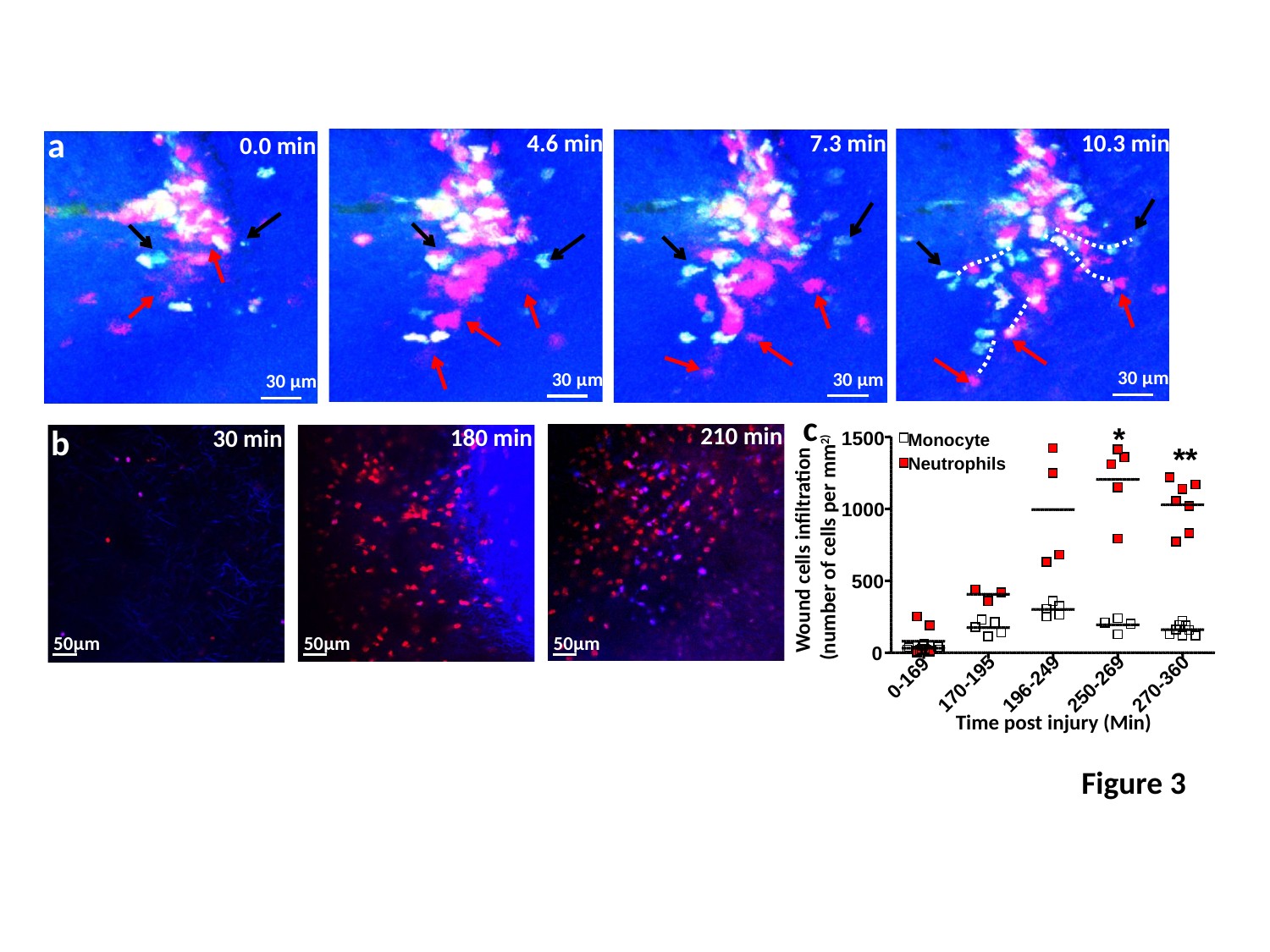

a
30 μm
30 μm
30 μm
30 μm
4.6 min
7.3 min
10.3 min
0.0 min
1500
Monocyte
Neutrophils
1000
500
0
0-169
170-195
196-249
250-269
270-360
Time post injury (Min)
Wound cells infiltration
 (number of cells per mm2)
c
210 min
b
180 min
30 min
50μm
50μm
50μm
**
Figure 3
*

## Slide 4
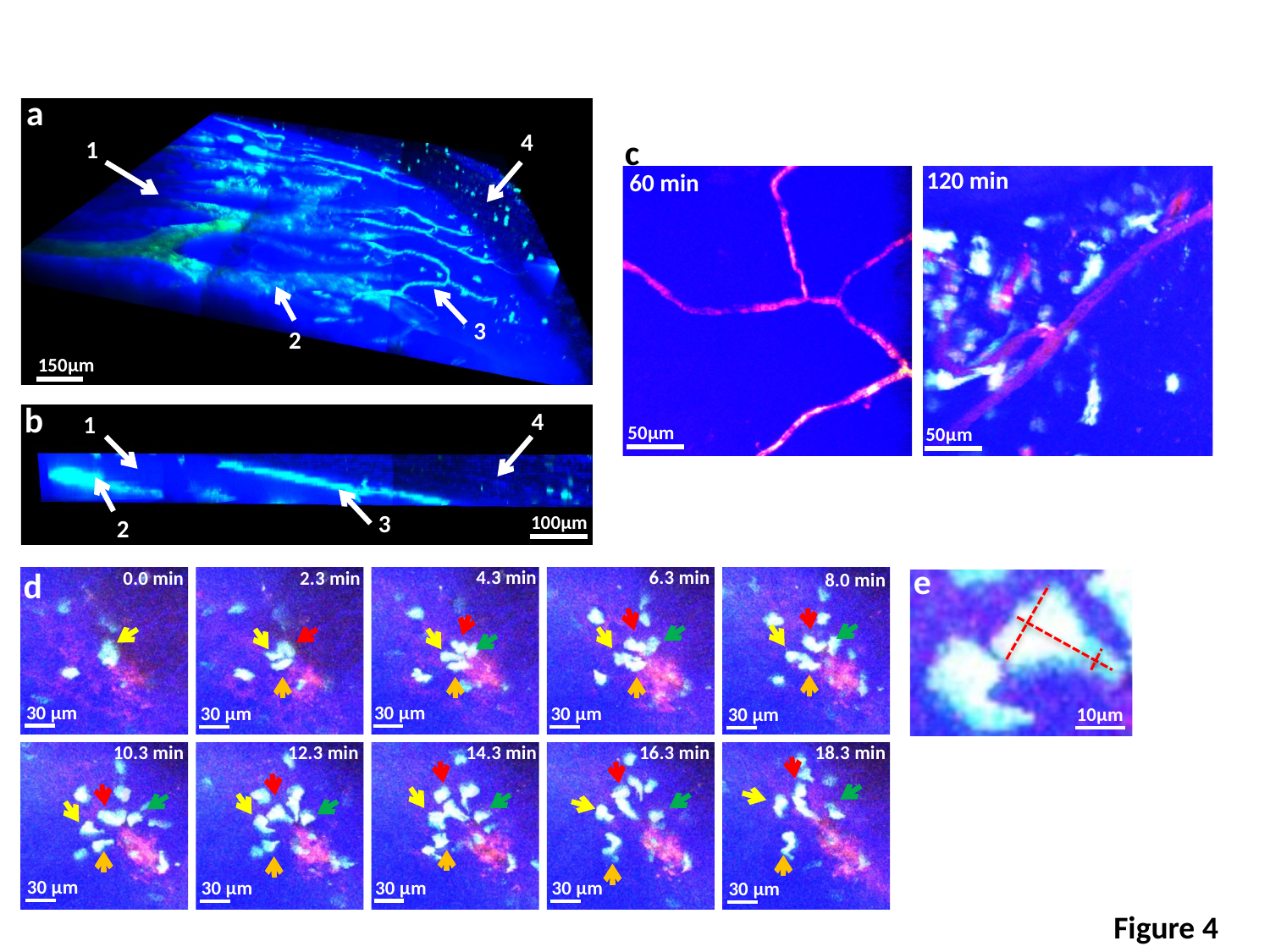

a
4
1
3
2
c
120 min
60 min
50μm
50μm
150μm
b
4
1
3
100μm
2
e
d
30 μm
30 μm
30 μm
30 μm
30 μm
30 μm
30 μm
30 μm
30 μm
30 μm
4.3 min
6.3 min
0.0 min
2.3 min
8.0 min
10μm
14.3 min
16.3 min
10.3 min
12.3 min
18.3 min
Figure 4

## Slide 5
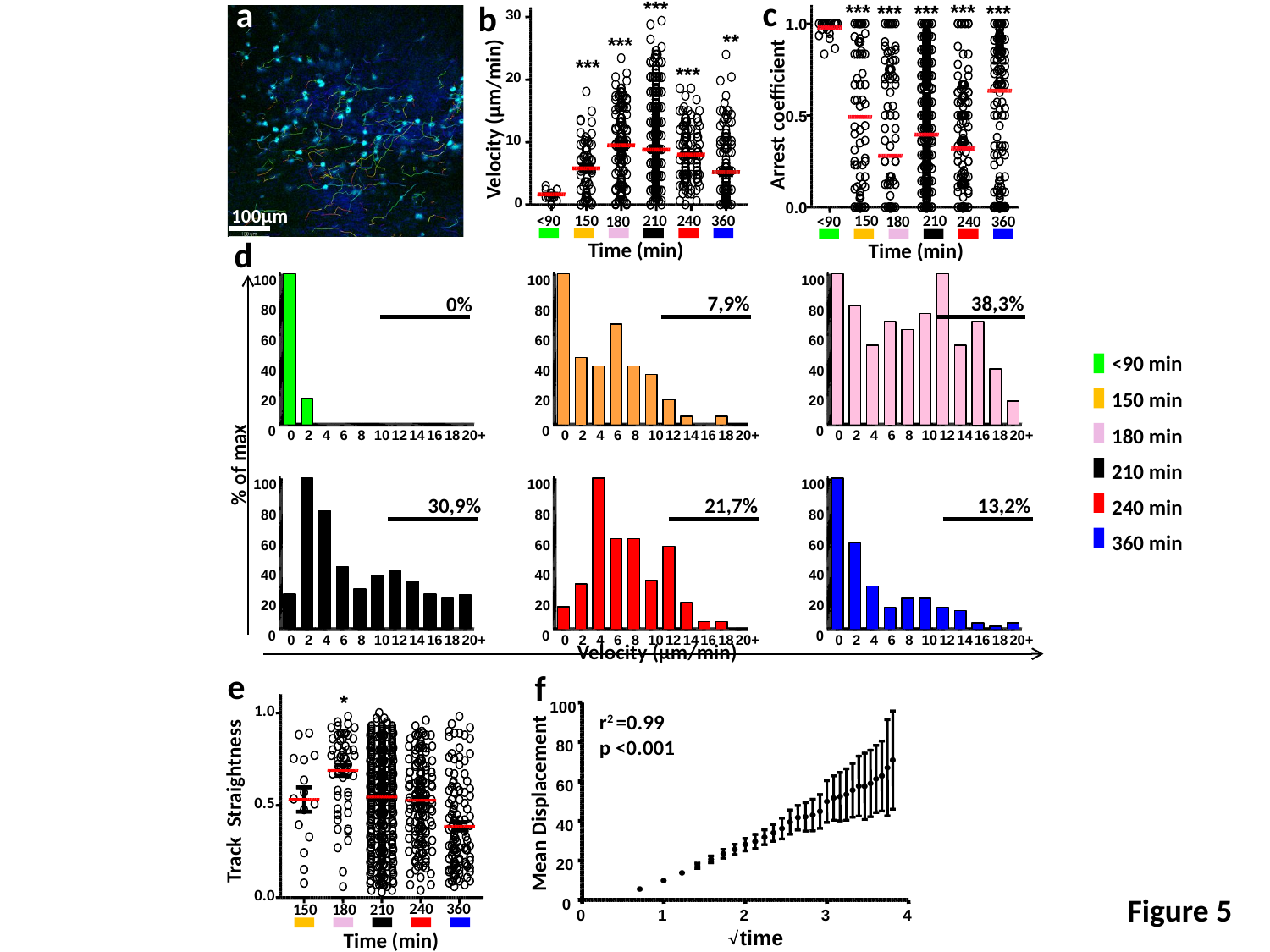

c
1.0
Arrest coefficient
0.5
0.0
150
210
240
360
<90
180
a
b
30
20
Velocity (µm/min)
10
0
150
210
240
360
<90
180
Time (min)
***
***
***
***
***
**
***
***
***
100μm
d
100
0
2
4
6
8
10
12
14
16
18
20+
80
60
40
20
0
100
0
2
4
6
8
10
12
14
16
18
20+
80
60
40
20
0
0
2
4
6
8
10
12
14
16
18
20+
80
60
40
20
0
100
100
0
2
4
6
8
10
12
14
16
18
20+
80
60
40
20
0
100
0
2
4
6
8
10
12
14
16
18
20+
80
60
40
20
0
100
0
2
4
6
8
10
12
14
16
18
20+
80
60
40
20
0
7,9%
38,3%
0%
30,9%
21,7%
13,2%
% of max
Velocity (µm/min)
Time (min)
<90 min
150 min
180 min
210 min
240 min
360 min
e
*
240
360
150
180
210
1.0
Track Straightness
0.5
0.0
Time (min)
f
100
 r2 =0.99
 p <0.001
80
60
Mean Displacement
40
20
0
0
1
2
3
4
time
Ö
***
Figure 5
