# Supplementary material for: Correction: In Vivo Imaging Reveals a Pioneer Wave of Monocyte Recruitment into Mouse Skin Wounds
Source: PLoS One. 2014 Dec 8;9(12):e115508. doi: 10.1371/journal.pone.0115508 (PMC4259483; doi:10.1371/journal.pone.0115508)
Supplement: Figure S1 — GFP+ ECFP- cells were detectable within the skin surrounding the wound. Representative TPLSM pictures of superficial skin layer from MacBlue ×CX3CR1gfp/+ mice at proximity of the wound edge. SHG signal is in blue, GFP signal is in green. (PPTX) [file pone.0115508.s001.pptx]

## Slide 1
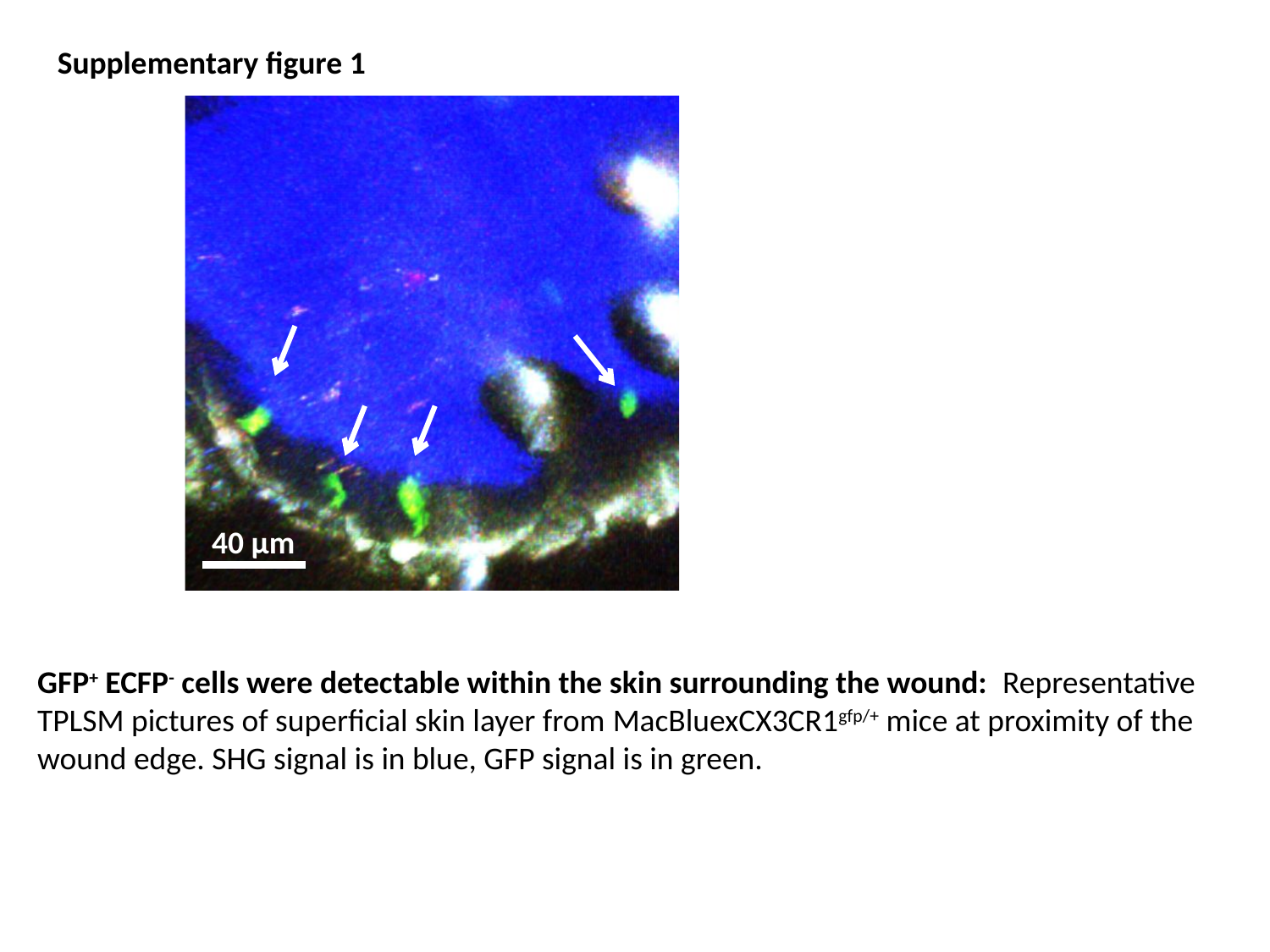

Supplementary figure 1
40 µm
GFP+ ECFP- cells were detectable within the skin surrounding the wound: Representative TPLSM pictures of superficial skin layer from MacBluexCX3CR1gfp/+ mice at proximity of the wound edge. SHG signal is in blue, GFP signal is in green.
